# Patterns of cortical activity during the observation of Public Service Announcements and commercial advertisings

**DOI:** 10.1186/1753-4631-4-S1-S3

**Published:** 2010-06-03

**Authors:** Giovanni Vecchiato, Laura Astolfi, Febo Cincotti, Fabrizio De Vico Fallani, Domenica M Sorrentino, Donatella Mattia, Serenella Salinari, Luigi Bianchi, Jlena Toppi, Fabio Aloise, Fabio Babiloni

**Affiliations:** 1 IRCCS "Fondazione Santa Lucia", Rome, Italy; 2 Department of Human Physiology and Pharmacology, University “Sapienza”, Rome, Italy; 3 Department of Informatica e Sistemistica, University “Sapienza”, Rome, Italy; 4 Dept of Neuroscience, University “Tor Vergata”, Rome, Italy

## Abstract

**Background:**

In the present research we were interested to study the cerebral activity of a group of healthy subjects during the observation a documentary intermingled by a series of TV advertisements. In particular, we desired to examine whether Public Service Announcements (PSAs) are able to elicit a different pattern of activity, when compared with a different class of commercials, and correlate it with the memorization of the showed stimuli, as resulted from a following subject’s verbal interview.

**Methods:**

We recorded the EEG signals from a group of 15 healthy subjects and applied the High Resolution EEG techniques in order to estimate and map their Power Spectral Density (PSD) on a realistic cortical model. The single subjects’ activities have been z-score transformed and then grouped to define four different datasets, related to subjects who remembered and forgotten the PSAs and to subjects who remembered and forgotten cars commercials (CAR) respectively, which we contrasted to investigate cortical areas involved in this encoding process.

**Results:**

The results we here present show that the cortical activity elicited during the observation of the TV commercials that were remembered (RMB) is higher and localized in the left frontal brain areas when compared to the activity elicited during the vision of the TV commercials that were forgotten (FRG) in theta and gamma bands for both categories of advertisements (PSAs and CAR). Moreover, the cortical maps associated with the PSAs also show an increase of activity in the alpha and beta band.

**Conclusions:**

In conclusion, the TV advertisements that will be remembered by the experimental population have increased their cerebral activity, mainly in the left hemisphere. These results seem to be congruent with and well inserted in the already existing literature, on this topic, related to the HERA model. The different pattern of activity in different frequency bands elicited by the observation of PSAs may be justified by the existence of additional cortical networks processing these kind of audiovisual stimuli. Further research with an extended set of subjects will be necessary to further validate the observations reported in this paper.

## Background

The aim of the brain imaging techniques applied to the fruition of commercial advertising is to understand mechanisms underlying customer’s engagement with brand or company advertised [1-3]. In particular, the issue is to explain how the exposure of subsequent film segments are able to trigger in the consumer mind persisting stimuli leading to an interest, preference, purchase and re-purchase of a given product. In the last decades, several Authors have investigated the capability of subjects to memorize and retrieve sensible “commercial” information observed during a TV spot [4-9].

Local governs of European countries, and also across the world, are called to disseminate information about health risky habits, promoting instead healthy life style, in order to increase the health of their citizens. In this context Public Service Announcements (PSAs) are non-commercial broadcasted ads intended to modify public behaviour. PSAs are at the core of many public health campaigns against smoke, fatty foods, abuse of alcohol and other possible threats for the health of citizens. But the content of these PSAs could be also directed for the promotion of “positive” social collective behaviour, such for instance against racism, supporting the integration of different cultures in the country, or for instance promoting a healthy drive style, for the road security. When effective, PSAs carry a great public health benefit [10,11]. However, the lack of reliable, quantitative and objective means of ad evaluation is one of the impediments to better PSA outcomes. In addition, not well designed PSAs are going to have counter effects with respect to their desired goals [12]. On the other hand, commercial advertisings are announcements of material or goods of potential interest for the public, usually offered for the purchase. So while the two kind of announces are often similar for realization, they are different for the message convoyed to the public.

The purpose of this paper is to illustrate the potential of the High Resolution EEG techniques, when applied to the analysis of brain activity related to the observation of TV commercials and PSAs, to localize cerebral areas mostly emotionally involved. In particular, we would like to describe how, by using appropriate statistical analysis, it is possible to recover significant information about cortical areas engaged by particular scenes inserted within the video clip analyzed. The brain activity was evaluated in frequency domain by solving the associate inverse problem of EEG with the use of realistic head models. Successively, the data analysed were statistically treated by comparing their actual values to the average values estimated during the observation of the documentary. Statistical estimators were then evaluated and employed in order to generate representations of the cortical areas elicited by the particular video considered. The present study has been performed by providing subjects with a comfortable environment while they were watching the TV screen, without particular thoughts and requirements in their mind. In fact, subjects were simply ask to observe the movie we proposed without any mention about the verbal interview that would have been performed later, about two hours after the end of the task.

With the particular task adopted and for the analyzed population, we may summarize the experimental questions of the present study as follows:

1. Are there particular EEG activities in the spectral domain which correlate with the memorization of PSAs and CARs TV advertisements?

2. Is it possible to discriminate different patterns of activity by comparing the EEG cortical spectral maps associated with the observation of PSAs and CARs commercials? 	

## Methods

### Experimental design

Fifteen healthy volunteers (mean age 27,5±7,5 years; 7 women) have been recruited for this study. The procedure of the experimental task consisted in observing a thirty minutes long documentary in which we inserted three advertising breaks: the first one after eight minutes from the beginning, the second one in the middle and the last one at the end of the movie. Each interruption was formed by the same number of commercial videoclips of about thirty seconds. During the whole documentary, a total of sixteen TV commercials were presented. The clips were related to standard international brands of commercial products, like cars, food, etc. and public service announcements (PSAs) such as campaigns against violence. In particular, a PSA and a car advertising was showed in each commercial break. In this way, each experimental subject was exposed to the observation of a total of three PSA and three commercials about cars. In the present analysis we took into account only the cerebral activity associated to the observation of PSA and cars (CAR) videoclips. Randomization of the occurrence of the commercial videos within the documentary was made to remove the factor “sequence” as possible confounding effect in the following analysis. During the observation of the documentary and TV commercials, subjects were not aware that an interview would be held within a couple of hour from the end of the movie. They were simply told to pay attention to what they would have watched and no mention of the importance of the commercial clips was made. In the interview, subjects were asked to recall commercial clips they remembered. According to the information acquired, the neurophysiological activity recorded has been divided into four different datasets. The first pool was related to the activity collected during the viewing of the PSA (CAR) clips that the subjects had correctly remembered, and this dataset was named PSA_RMB_ (CAR_RMB_). The second pool was related to the activity collected during the observation of the PSA (CAR) that had been forgotten by the subjects, and this set was named PSA_FRG_ (CAR_FRG_). In such a way, the four different EEG datasets considered in this study are PSA_RMB_, PSA_FRG,_ CAR_RMB,_ CAR_FRG_ which refer to our four different experimental conditions. Finally, the electroencephalographic activity elicited during the observation of the documentary was also analyzed and a final pool of data, related to this state, was generated with the name REST. This REST period was taken as the period in which the subject looked at the documentary. We took into account a two minutes long sequence of the documentary, immediately before the appearance of the first spot interruption, employed in order to minimize the variations of the spectral responses owing to fatigue or loss of concentration.

### Cerebral recordings

The cerebral activity was recorded by means of a portable 64-channel system (BE+ and Galileo software, EBneuro, Italy). Informed consent was obtained from each subject after explanation of the study, which was approved by the local institutional ethics committee. All subjects were comfortably seated on a reclining chair, in an electrically-shielded, dimly-lit room. Recordings were initially extra-cerebrally referred and then converted to an average reference off-line. We collected the EEG activity at a sampling rate of 256 Hz while the impedances kept below 5 kΩ. Each EEG trace was then converted into the Brain Vision format (BrainAmp, Brainproducts GmbH, Germany) in order to perform signal pre-processing such as artefacts detection, filtering and segmentation. Raw EEG traces were first band pass filtered (high pass = 2 Hz, low pass = 47 Hz) and the Independent Component Analysis (ICA) was then applied to detect and remove components due to eye movements, blinks, and muscular artefacts. The ICA returned a set of EEG traces that were free from the artifacts induced by the natural eyeblinks that occurred during the visualization of the commercials. In addition, ICA removed appropriately as well as from the muscular artefacts that occurs during the behavioural adjustments along the observation of the documentary.

These EEG traces were then segmented to extract the cerebral activity during the observation of the TV commercials and that associated to the REST period. These resulting segments have been used to evaluate the cortical activity and calculate the Power Spectral Density (PSD) for each segment.

All segments were exported in binary format and then converted for further data processing performed with in-house MATLAB software. The estimation of the power spectra at the cortical level was performed with our MATLAB software that was checked to reproduce identical results to the usual common MATLAB based systems, like BrainStorm or even cortical LORETA. Since the frontal areas have been depicted to play a clear role for the phenomena we would like to investigate [13,14], we used the frontal electrodes to compute the PSD used in the following of this study: the EEG signals were subjected to the computation of the Power Spectral Density by taking into account the signals that comes from the following frontal and prefrontal electrodes of the 10-10 International System: F3, F4, AF3, AF4, F7, AF7, F8, AF8, Fz, AFz.

### Estimation of cortical activity

In this work, cortical activity from EEG scalp recordings was estimated by employing the high-resolution EEG technologies [15-17] with the use of a realistic head model. This model consists of about 5,000 dipoles uniformly disposed on the cortical surface and the estimation of the current density strength for each dipole was obtained by solving the linear inverse problem according to techniques described in previous papers [18-20] and illustrated in the following.

The solution of the following linear system:

**Ax** = **b** + **n** (1)

provides an estimation of the dipole source configuration **x** that generates the measured EEG potential distribution **b**. The system also includes the measurement noise **n**, assumed to be normally distributed [17,21]. **A** is the lead field matrix, where each j-th column describes the potential distribution generated on the scalp electrodes by the j-th unitary dipole. The current density solution vector **ξ** of Eq. 1 was obtained as [21]:

 (2)

where **M**, **N** are the matrices associated with the metrics of the data and of the source space, respectively, λ is the regularization parameter and || **x** ||_M_ represents the M norm of the vector **x**. The solution of Eq. (2) is given by the inverse operator **G**:

,    (3)

A regularization of this linear system was obtained by the standard approach proposed by the Brainstorm toolbox [22] which sets the regularization parameter as the ten percent of the maximum eigenvalue of the matrix defined by the product . As a metric in the data space we used the identity matrix (i.e. M = I), while as a norm in the source space we used the following metric:

 (4)

where (**N**^-1^)_ii_ is the i-th element of the inverse of the diagonal matrix **N** and all the other matrix elements N_ij_ are set to 0. The L_2_ norm of the i-th column of the lead field matrix **A** is denoted by ||**A_.i_**||.

### Statistical spectral analysis

In order to calculate the PSD for each segment and then transform the cortical activity into Z variables, we employed the following mathematical procedure.

Let: 

 (5)

be the vector of scalp measurements in a given time window, with Δτ being the sampling time and the corresponding vector of cortical estimates given by Eq.2,  the Fourier transform of . We define the matrix of Cross-Power Spectral Densities (**CSD**) as the matrix whose element (i, j) is the cross-spectrum of i-th and j-th channel of the signal. By using the exponent ‘sens’ to indicate the sensors’ measurements and the exponent ‘src’ to indicate the cortical sources we have: 

 (6)

where the  is the conjugate transposed (Hermitian) of . Analogously: 

 (7)

By using Eq.3 we obtain:

  (8)

If  and  are not deterministic signals, but rather we have several trials (realizations) of a stochastic process, Eq (8) holds if we substitute **CSD**s with their expected values or with their estimates (i.e. ).

In case we were interested in calculating the (Auto-)Power Spectral Densities (PSDs) of estimated cortical sources, we only need to compute the diagonal of :

	 (9)

where the variable j indicates the number of sources.

The spectral resolution of this method is inversely proportional to .

The level of noise in the EEG linear inverse solutions can be addressed by estimation of the ‘projection’  of the EEG noise  onto the cortical surface by means of the computed pseudoinverse operator **G** (as described in Eq. 3); the variance of the noise on the estimated source strength  is given by 

 (10)

where  is the j-th row of the pseudoinverse matrix,  is the EEG noise covariance matrix (), and  is the expectation operator. A common choice for the estimation of the sensor noise covariance matrix is to select an interval of data (baseline) where no task-related activity is supposed to occur and thus all signals are believed to be noise:

 (11)

According to the DSPM approach (Dale et al 2000), the following normally-distributed cortical z-score estimator can be obtained for each j-th cortical location and for each time point considered:

  (12)

This allows us to assess quantitatively the ratio between the estimated cortical activity  and the amount of noise at cortical level, quantified through the standard deviation of its estimate. Values of z exceeding a given threshold represent levels of estimated cortical activity that are unlikely owing to chance alone but are related to the task performed by the experimental subject. For instance, the threshold for the z-score level at 5% (uncorrected for multiple comparisons) is . 	

In this particular application, we considered as baseline the estimated cortical activity during the viewing of the documentary. Here, we extend the DSPM approach to the analysis of the power spectra variations during the experimental task. The computation of the z-score level in the spectral case is performed according to the following:

 (13)

where  indicates the variance of the estimate of the spectral density of the EEG measurements during the baseline period at the considered frequency f, and  is the z-score for the j-th current dipole at frequency f, while the inverse operator **G** is the same used for the temporal case. The  is a Z score variable for construction. Following these calculations it has been possible to obtain and analyse spectral Z variables for the canonical frequency bands of interests: Theta (4-7 Hz), Alpha (8-12), Beta (13-24), Gamma (25-40). We have to mention that spectral analysis is usually applied in the context of the stationary analysis of a EEG signals. In this particular case, since we are interested in the analysis of the cerebral activity during the whole observation of the remembered and forgotten videos, the possible non-stationarity of the EEG data (and the relative variation in time of the spectral profiles of the EEG activity) is not investigated here.

### Contrast between experimental conditions on the cortical spectral maps

We initially calculated statistical spectral maps for each subject, each PSA and CAR TV commercial, in the four frequency bands. Since we transformed the PSD data into Z variables, it has been possible to group the single subjects activities according to the answers which they gave in the memory questions during the interview. In this way the cerebral activity recorded during the observation of the advertisements has been considered as belonging to the groups PSA_RMB_, PSA_FRG,_ CAR_RMB,_ CAR_FRG_. The cortical maps depicting the statistical contrasts between the remembering (RMB) and forgetting (FRG) conditions were then generated for each frequency band of interest.

All the statistically-activated areas of each subject were mapped on a common cortical representation through such transformation. For display purposes, we represented the results obtained from the average brain model created with the BRAINSTORM software freely downloadable from internet [22]. The average brain model, however, is not used for the computations performed but just for the visualization of the population data. In particular, the average brain model was used to display the cortical areas statistically significantly activated during the different experimental conditions in all the subjects analyzed. Statistical significant activity will be presented according to the Bonferroni-Holm corrections for multiple comparisons at 5% of the significance level.

## Results

The Power Spectral Density has been calculated for all EEG segments, which are associated to the observation of the PSA and CARs advertisements, by means of the mathematical procedures described in the Methods section. For each subject, these PSDs have been evaluated for all the frequency bands of interest and then compared with the values of power spectral density elicited by the observation of the documentary (REST segment). The contrast between these two experimental conditions has been performed by estimating the z-score variables. In such a way, the cortical distributions of the z-scores related to the observation of the commercial videoclips have been organized into four different groups: the first two refer to the cerebral activity elicited by the observation of PSAs which have been remembered by the subjects during the interview (PSA_RMB_) and those which have been forgotten during the same interview (PSA_FRG_); in the same way, we grouped the cortical activity elicited by the observation of the CAR TV spot by separating that associated to the EEG segments of subjects who remembered such commercials (CAR_RMB_) from that associated to the EEG segments of subjects who forgotten such commercials (CAR_FRG_). Contrasts will be made among the cortical z-score distributions of the defined experimental groups by comparing the cerebral activities related to both the RMB and FRG conditions within the same category of commercials (PSA_RMB_ vs PSA_FRG_; CAR_RMB_ vs CAR_FRG_), and by comparing the same cerebral activities between the two categories of advertisements (PSA_RMB_ vs CAR_RMB_; PSA_FRG_ vs CAR_FRG_). The resulting statistical spectral maps of z-scores highlight the cortical areas in which the estimated power spectra statistically differ between the populations considered. The following maps refer to the contrast between the experimental populations in the theta (upper left), alpha (upper right), beta (lower left) and gamma (lower right) frequency bands.

### Statistical maps of the spectral cortical activity during the observation of the PSAs

**Figure 1:**
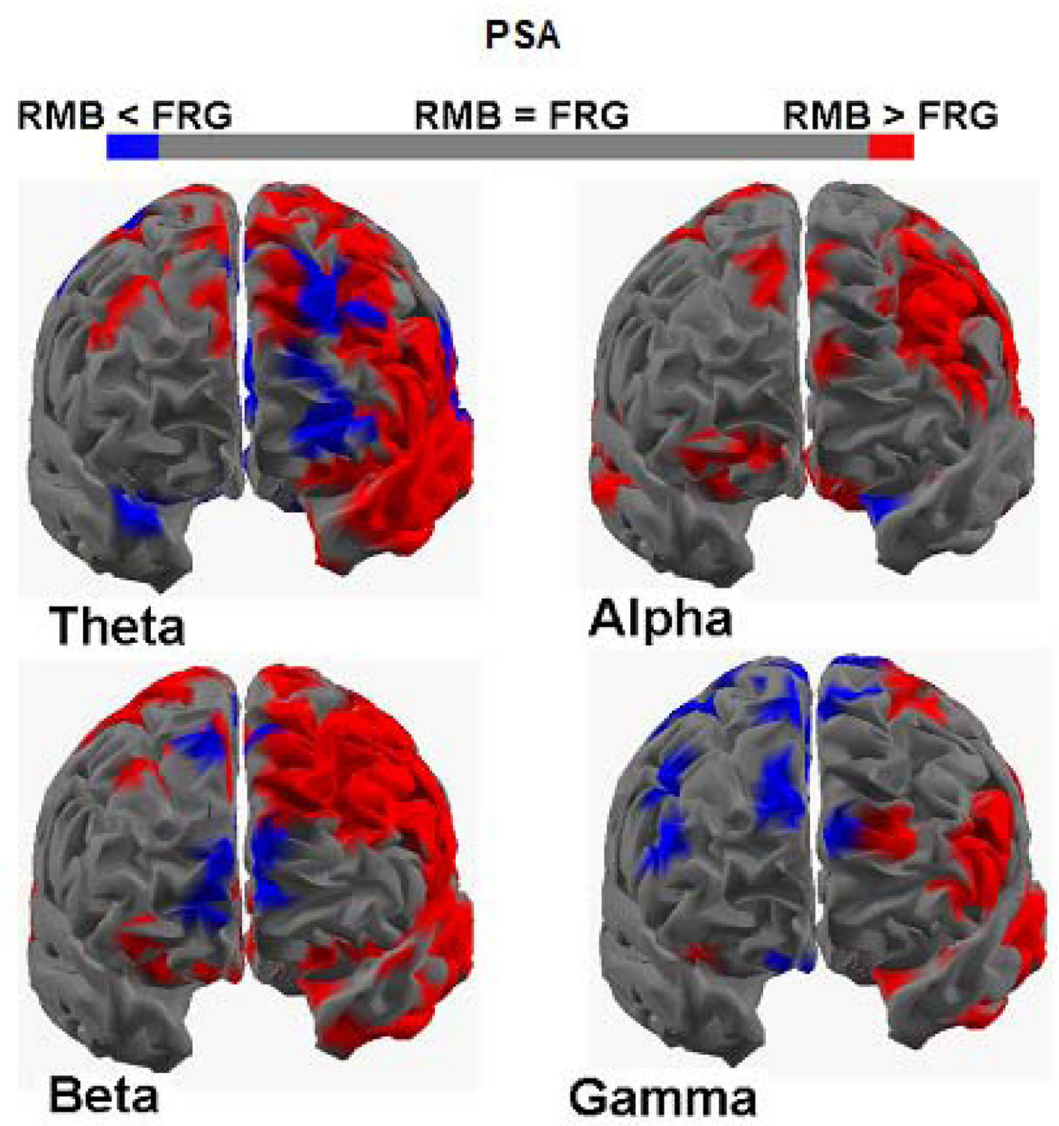
**Four cortical maps for the groups PSA_RMB_ and PSA_FRG_** Figure presents four cortical z-score maps, in the four frequency bands employed. Colour bar represents cortical areas in which increased statistically significant activity occurs in the PSA_RMB_ group (subjects watching PSAs that will be remembered) when compared to the PSA_FRG_ group (subjects watching PSAs that will be forgotten) in red, while blue is used otherwise (p < 0,05 Bonferroni-Holm corrected). Grey colour is used to map cortical areas where there are no significant differences between the cortical activity in the PSA_RMB_ and PSA_FRG_ groups.

Figure 1 presents four cortical maps in which the brain is viewed from a frontal perspective. The colour scale on the cortex coded the statistical significance: where there are cortical areas in which the power spectrum does not differ between the two populations, a grey colour was employed. The red colour was employed when the cortical areas present a statistically significance power spectral activity greater in the population that remembered the commercial videos (RMB) with respect to the other, while the blue colour coded the opposite situation (i.e. the power spectral activity in the group that forget the commercial videos is greater with respect to the brain activity in the group that remembers the ads).

Figure 1 shows an increase of cortical activity in all the bands of interest that is prominent on the left frontal hemisphere for the PSA_RMB_ group. In particular, the cerebral activity in theta band presents a statistically significant activation among frontal areas of the left lobule for the PSA_RMB_ group, although there are also some prefrontal areas depicted by the activation due to the PSA_FRG_ population. This sensible activation for the PSA_RMB_ dataset is still clearer by examining the spectral cortical activity in the alpha and beta bands: the cortical maps relating to these frequency bands are characterized by a statistically significant PSD values located in a wide area of the left frontal hemisphere. Instead, the spectral map of the gamma band presents both significant activations of activity for the PSA_RMB_ group and for the PSA_FRG_ population in cortical regions located in the left and in the right hemisphere of the frontal lobule respectively.

### Statistical maps of the spectral cortical activity during the observation of the CAR commercials

**Figure 2:**
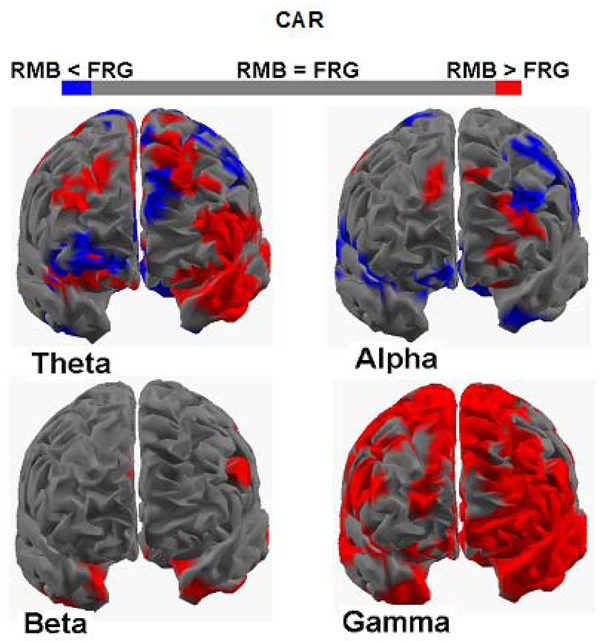
**Four cortical maps for the groups CAR_RMB_ and CAR_FRG_** Same conventions than in the previous figure except the use of red colour to code the cortical areas in which the brain activity of the CAR_RMB_ group (subjects watching commercials about cars that will be remembered) is significantly higher than the activity of the CAR_FRG_ group (subjects watching commercials about cars that will be forgotten). The blue colour is used to map brain areas in which the activity of the CAR_FRG_ group is significantly higher than the activity of the CAR_RMB_ group.

Figure 2 presents the contrast between the CAR_RMB_ and CAR_FRG_ groups in the four frequency bands considered in this analysis by using the same convention of Figure 1. The significant increase of the frontal activity in the theta band is clearly visible in the CAR_RMB_ group (in red) when compared to the CAR_FRG_ one (in blue), in the left upper part of the Figure 2. Scattered increased of cortical activity on the left hemisphere is also present in the CAR_FRG_ group. Instead, the alpha frequency map shows scattered increased activations for both CAR_RMB_ and CAR_FRG_ groups, bilaterally located in the left and right hemispheres. Similar considerations can be done for the spectral activity in the beta band since it presents only a few spots of significant activations for the CAR_RMB_ population. The cortical activity in the gamma band is greater in the CAR_RMB_ group which shows a large statistically significant activations spreading both in the pre and frontal regions of the left hemisphere and in the frontal areas of the right hemisphere.  

## Discussion

Taken together, the results obtained in this study indicated the cortical activity in the theta band on the left frontal areas was increased during the memorization of commercials. Such results are then congruent with the role that has been advocated for the left pre and frontal regions during the transfer of sensory percepts from the short term memory toward the long-term memory storage by the HERA model [13,14,24,25]. In fact, in such model, the left hemisphere plays a key role during the encoding phase of information from the short term memory to the long term memory, whereas the right hemisphere plays a role in the retrieval of such information. 

Moreover, apart from the specific activation of the prefrontal left cortices (as expressed in the increase of the spectral power in the theta and gamma bands) we also found out an enhance of activity in different part of the cortex in the alpha and beta bands among the population who remembered the PSAs we proposed. This result is not evident by inspecting the cortical maps associated to the CARs. This fact could be interpreted by assuming the existence of different cortical areas processing these two different kinds of audiovisual stimuli. In fact, we could speculate that the fact that the PSAs are related to issues treating health and possible threats for the individual could elicit an increased level of attention when compared to the observation of standard CARs. Such increased level of attention could be reflected by the increase of the EEG spectral power in the upper frequency bands, often linked to the increase of aspecific attention to the external world [26-28]. The different cortical areas elicited seems hence to be a property of PSAs when compared to the CARs.

## Conclusions

The results of the present study suggested the following answers to the experimental questions exposed in the Background section:

“ The results of the present study suggested the following answers to the experimental questions exposed in the Background section in the population analyzed;

1) there is a difference in cerebral activity during the observation of commercials and PSAs that will be memorized when compared to the cerebral activity during the observation of commercials and PSAs that will be forgotten. This difference is localized in particular frequency bands, in particular to the theta band and gamma bands. Such differences are also related to particular cortical areas involved in the encoding of new information (i.e left frontal hemisperes).

2) There are differences in brain activity during the observation of commercials related to car and PSAs; such differences are related to the alpha and beta frequency bands.

In conclusion, the TV commercials proposed to the population analyzed have increased their cerebral activity mainly in the theta and gamma band in the left hemisphere, when they will be memorized, for both categories of advertisements. In addition, the memorization of PSAs activated a different cortical distribution of the power spectra of the EEG in the alpha and beta frequency bands. Further research with an extended set of subjects will be necessary to further validate the observations reported in this paper, since the size of the experimental population here employed could lower the statistical power of the conclusions here reported. However, these conclusions seems reasonable and well inserted in the already existing literature on this topic related to the HERA model.

## Competing interests

There are no competing interests (financial, political, personal, religious, ideological, academic, intellectual, commercial or any other) to declare in relation to this manuscript.

## Authors' contributions

Giovanni Vecchiato performed the data analysis and participated to the generation of the experimental design, together Febo Cincotti. Dr. Vecchiato also wrote the draft of the present paper. Dr. Laura Astolfi and Fabrizio De Vico Fallani provides the data analysis from a set of artifact-free trials collected. Dr. Donatella Mattia and Ramon Soranzo planned the experimental design and participated to the evaluation of the results. Domenica Maria Sorrentino performed the measurements and revised the EEG traces. Luigi Bianchi wrote the software for the data analysis and participated to the discussion of the data. Prof. Babiloni participated to the data analysis and revised the draft version of the manuscript.
